# Prototype of a New Head Grabber for Robotic Strawberry Harvesting with a Vision System

**DOI:** 10.3390/s24206628

**Published:** 2024-10-14

**Authors:** Zygmunt Sobol, Sławomir Kurpaska, Piotr Nawara, Norbert Pedryc, Grzegorz Basista, Janusz Tabor, Tomasz Hebda, Marcin Tomasik

**Affiliations:** Faculty of Production and Power Engineering, University of Agriculture in Krakow, ul. Balicka 116 B, 30-149 Krakow, Poland; zygmunt.sobol@urk.edu.pl (Z.S.); slawomir.kurpaska@urk.edu.pl (S.K.); piotr.nawara@urk.edu.pl (P.N.); grzegorz.basista@urk.edu.pl (G.B.); janusz.tabor@urk.edu.pl (J.T.); tomasz.hebda@urk.edu.pl (T.H.); marcin.tomasik@urk.edu.pl (M.T.)

**Keywords:** end-effector, sensors, algorithm, strawberry, vision system

## Abstract

This paper presents the design of a strawberry fruit head gripper unit, together with the concept of a control system for the operation of its mechanisms and vision system. The developed design consists of three specialised mechanisms: positioning, grasping, and cutting off of the fruit. A Finite Element Method (FEM) model was developed for the described design. Next, calculations were carried out, based on which the construction materials were selected. The key performance parameters of the functional model, built on the basis of the developed design concept, were verified under laboratory conditions. In tests carried out on the possible hematoma caused by exceeding the breaking stress induced by the pressure of the encompassing jaws on the fruit, it was found that none of the fruit tested suffered mechanical damage as a result of the sensor triggering force, and the average length of the trimmed stalk was approximately 14 mm. The designed head gripper, together with the proposed automation system, will contribute to improving harvesting precision, and this will favour a reduction in the quantitative and qualitative losses of the harvested crop. The experimental tests conducted under harvesting conditions showed a high efficiency of 95% in identifying ripe fruit, and the harvesting efficiency of the robotic arm was 90%.

## 1. Introduction

The increasing global population is increasing the demand for fresh fruits and vegetables. In light of qualified labour shortages in agriculture, especially in harvesting, agriculture faces challenges in terms of obtaining an adequate quantity and quality of food products. Obtaining a certain quality of harvested fruit or vegetables also depends on ensuring a correct harvesting process. Hence, intensive research on the robotisation of this process is underway in numerous research centres. Attempts at robotic harvesting have been made, for example, for harvesting peppers in greenhouse cultivation [1], cucumbers [2,3], tomatoes [4,5], and other fruit, including kiwifruit [6], apples [7], and citrus fruit [8]. Li et al. [9] proposed an improved YOLOv5s model for pitaya fruit detection in day and night environments with the addition of light, which was successfully implemented in an Android device. As for the design of the robot, Jun et al. [10] proposed that the work units for robotic fruit harvesting are divided into three main units: vision systems, a manipulation unit with fruit-harvesting components, and a gripper.

Strawberries are one of the most widely grown fruits in the world, not least because of their health-promoting qualities. For example, in 2022, 3.4 million tonnes of strawberries were produced in China and 1.3 million tonnes in the United States, providing the Carolinas alone with an annual revenue of USD 2 billion. Meanwhile, in Poland, strawberry production reached over 199,000 tonnes [11].

Unfortunately, due to the lack of people willing to work in strawberry picking, there is a need for the rapid development of systems to support this activity (such as autonomous transport trucks), as well as autonomous systems and robots, which would eliminate the need for human involvement in this activity. In the USA, for example, almost 70% of operators in the strawberry industry have invested in fully robotic strawberry-harvesting systems. A great deal of research has also been carried out in this area in research centres around the world. Thus, for example, Dimeas et al. [12], in order to reduce damage during robotic strawberry picking, proposed a hierarchical way of controlling the force of the gripper elements, concluding that the proposed solution—together with sensors to assess the pressure profile of the fruit—makes it possible to detect the misplacement of the gripper, and that the gripping efficiency is comparable to that of the human hand. Yamamoto et al. [13] presented the results of tests of a gantry robot dedicated to harvesting strawberries grown in Nutrient Film Technique (NFT) polytunnels. Xiong et al. [14] tested a proprietary robot and demonstrated its efficiency, of approximately 96%, in fruit harvesting. The total time to harvest one fruit ranged from 7.5 to 10.6 s.

**Figure 1:**
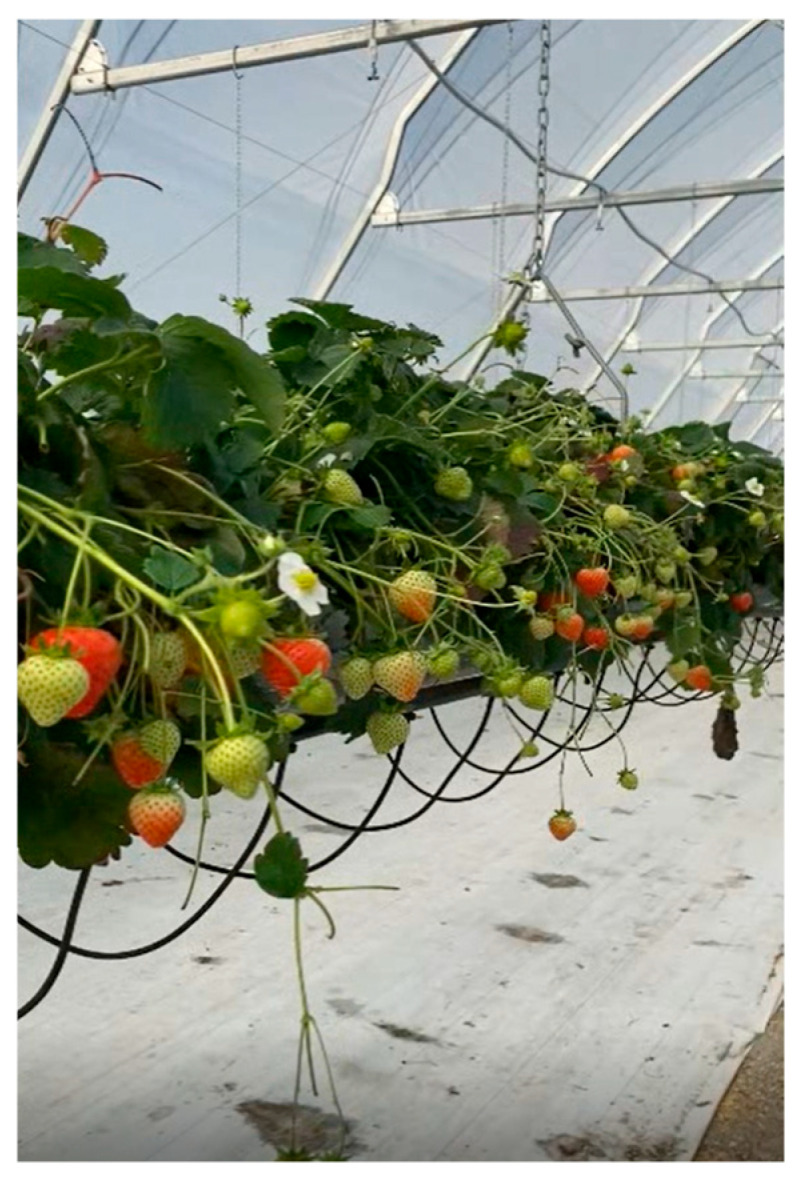
Example of fruit distribution (based on fruit availability).

The operation of the gripper heads themselves must meet a number of requirements that arise, among other things, from the operating environment in which the picker is to work (Figure 1). In the case of strawberries grown in NFT tunnels, the following scenarios should be taken into account:

- The fruits are exposed in such a way that the harvester unit is able to reach them and harvest them without causing any mechanical damage to the fruits themselves or to any unharvested fruits;
- The fruits are located in the top layer of the plant, but are positioned too close to each other, which could lead to the deterioration of the working conditions for the harvesting unit;
- The fruits are located in such a position (inside the plant) that it is almost impossible for the harvesting unit to reach them without damaging the fruits themselves or other unharvested fruits in the immediate vicinity.

**Figure 2:**
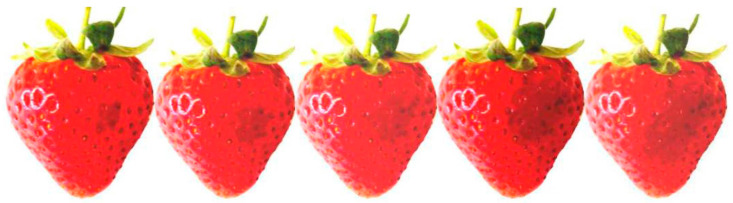
Images highlight the area of hematoma.

As a result of the impact of the working elements of the harvesting head (gripper) on the fruit tissue, wet or dry bruises occur once the tissue’s stress threshold is exceeded (Figure 2). Indentations can also easily result from contact with the surface of the fruit during hand picking. They are classified into the following two categories:

− Dry bruises—formed during harvesting or packaging, which are not progressive;
− Wet bruises—caused by damage due to rough handling of the fruit or by abrasion, which are progressive and can lead to further damage.

The presented review of the current state of research indicates that designing a harvesting head for autonomous agricultural robots remains an active area of inquiry. Accordingly, the aim of the study was to design a picker for strawberry harvesting that meets the following requirements:

- It enables contactless harvesting of fruit, thereby minimizing the risk of damage (hematoma).
- It effectively cuts the fruit from the stalk with a high degree of repeatability, leaving the peduncle length within the range of 5–20 mm, which can be adjusted according to the preferences of strawberry consumers.
- It incorporates structural features that allow it to reach and properly harvest the fruit located in the inner structure of the bush.
- It does not obstruct the vision system responsible for navigating the movement of its components to access the fruit to be collected.

Due to the fact that this analysis focuses solely on the operation of the prototype gripper, operational aspects, including the impact of harvesting conditions on the durability of its components, are not addressed in this paper.

## 2. Evaluation of the Pick-Up Head Gripper Concept

The design of a harvesting head specifically for soft fruit harvesting robots was developed using the morphological method, also known as the morphological card method, construction card method, or morphological box method. This method is primarily employed to stimulate creativity and generates new ideas, whether for developing new products or improving an existing ones. It serves as a standard and objective approach for evaluating designs within the industry [15].

One of the most important stages in the project execution according to the morphological method is the definition of design requirements. In the analysis presented in this paper, these requirements were established through the collaboration of experts from Cracow universities (AGH University of Science and Technology and Agricultural University of Cracow), as well as representatives of the strawberry producer, the Amplus company. The expert team comprised specialists in machine design, mechanics and operation, and strawberry cultivation.

In total, 13 criteria were used to evaluate the existing heads designed for robots that are employed in the strawberry harvesting process. These criteria were grouped into four categories: design features evaluation, operational technology, periodic maintenance, and economic factors. The first two categories were assigned a score of 40 points each by the experts, while the latter two received 10 points each. Individual design solutions were assessed against all criteria, receiving scores accordingly. The optimal design solution for each proposed harvesting head solution was determined based on Equation (1).

(1) $$\sum_{i=1}^{n}=V\cdot R$$

where:

V—the validity,
R—rating,
n—number of criteria selected analysis,
i—criterium.

Based on existing solutions in the literature and industry for harvesting heads designed for agricultural robots, we evaluated 11 structural designs. Among these, three designs ([16,17] (the same project) and [18,19]) use hinged jaws that create a chamber to encompass the harvested fruit in a non-invasive manner. Two solutions [14,17,19] include a mechanism for cutting the peduncle, while the design described in [18] harvests fruit by detaching the peduncle. The analysis indicates that the operational principle of five different solutions [20,21,22,23,24,25,26,27,28] relies on grippers that directly grasp the fruit, which interferes with the fruit tissue. Two designs [19,26] incorporate a vacuum pneumatic system, featuring a suction cup to harvest ripe fruit. This system also facilitates repositioning and extracting from the bush, allowing the working head to avoid harvesting impurities, such as torn leaves, unripe fruit, litter fragments, or soil clumps. The remaining three designs [28,29,30] employ jaws that grip the harvested fruit by the peduncle. Among these, two solutions [28,29] utilize a sliding jaw mechanism, while the design referenced in [30] operates using a scissor mechanism. The advantages of the recommended designs that grip the fruit by the peduncle include high ratings in several key features: structural compactness, slenderness (allowing easy access to the fruit or peduncle), minimal mechanical damage to the fruit, no contamination of the fruit crop, and reliable performance that considers the environmental impact during harvesting operations.

## 3. Concept of the Harvesting Head

**Figure 3:**
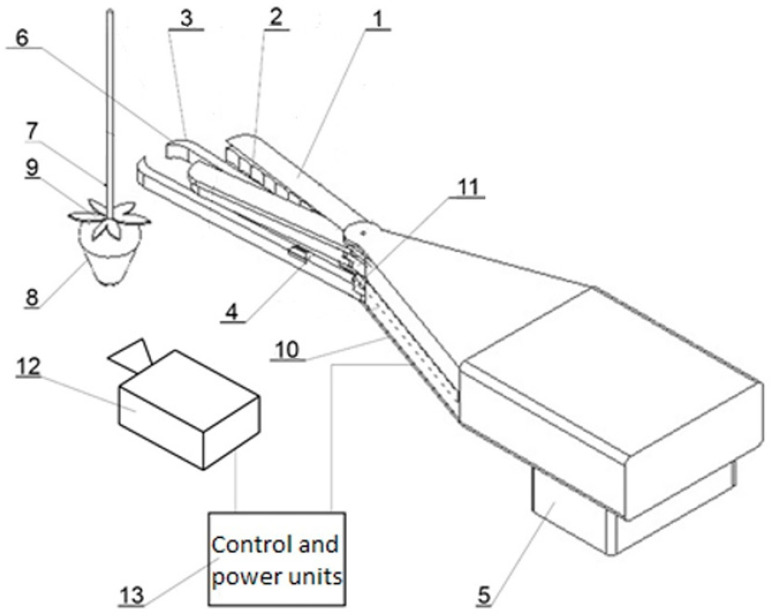
The scheme of a harvesting unit with a fruit displacement sensor located between the hinge enclosing the peduncle and the gripping jaws: 1—cutter, 2—clamping jaws, 3—gripping jaws, 4—sensor determining stalk length, 5—robot arm mount, 6—gripping jaw indentation, 7—stalk, 8—strawberry fruit, 9—flower, 10—harvesting assembly, 11—swinging and sliding mounting of the gripping jaw, 12—vision unit (camera), 13—central unit.

After the analysis, the authors proceeded to develop a novel and innovative harvesting head for strawberry fruit. Figure 3 illustrates the structural design of the head.

The designed strawberry fruit harvesting unit (10) is attached to a movable robotic arm (5) and consists of a body and three primary elements: gripping (3), clamping (2), and cutting (1) units. The innovative element of the designed harvesting head (10) is the gripping unit (3), which extends forward relative to the other elements of the head. This design allows for better penetration into the bush and pulls the stalk into the working range of the other working elements of the head. It comprises two gripping arms (3), at least one of which has an internal recess (6). When the arms close, an elongated opening is formed between them, allowing the stalk (7) to slide inside the gripping jaws (3). This assembly (3) also includes a displacement sensor (4), which functions to determine a fixed, preset length of the stalk to be cut off. The upward movement of the gripping arms (3), needed to make contact with the sensor terminals (4), is made possible by the pendulum mounting (11) of the gripping unit’s axis (3). The harvesting unit also includes the clamping unit (2), which consists of two jaws that clamp the stalk (7) of the fruit. The surfaces of the clamping jaws are parallel and, in the clamped position, are spaced from each other by a distance less than the thickness of the peduncle, thus ensuring stable retention of the peduncle with the fruit (8) during the cutting and transporting of the fruit to the container.

The cutting elements of the cutting unit (1) utilize a classic scissor mechanism. The cutting line of the cutting unit (3) is positioned at a certain distance from the upper surface of the sepals of the strawberry flower (9). This distance is adjustable from 5 mm to 30 mm and is equal to the sum of the thickness of the jaws of the gripping unit (3), the clamping unit (2), and half the thickness of the cutting unit (1), as well as the amount of play occurring between these elements.

Autonomous operation of the head is made possible by video acquisition from cameras (12), which send a data stream to the control unit (13). This unit analyses the camera data and indicates which fruit should be picked. In order to pick the fruit, a control signal is sent to the arm motors and the individual components of the harvesting head (10).

**Figure 4:**
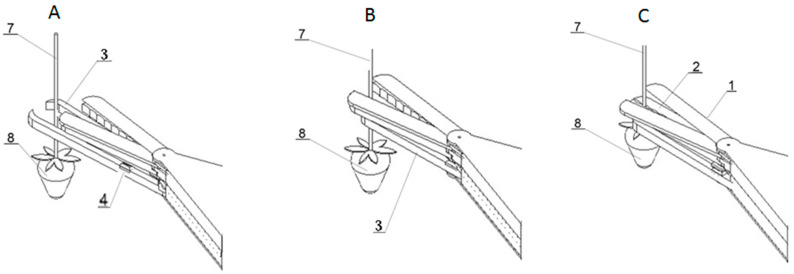
Harvesting stages: (**A**)—all the jaws are open, (**B**)—gripping jaws close, (**C**)—clamping jaws close, cutting elements begin to operate; 1—cutting elements, 2—clamping jaws, 3—gripping jaws, 4—sensor determining stalk length, 7—stalk, 8—strawberry fruit.

Figure 4 shows the successive phases of the harvesting head operation. Figure 4A shows the open jaws of the picker (3) extended towards the strawberry fruit (8). The clamping jaws (2) and cutting elements (1) are also open. The sensor contacts (4) detecting the movement of the fruit (8) remain in the ‘open’ position (no signal). The peduncle (7) of the fruit (8) is in the action zone of the gripping jaws. The gripping unit (3) is ready to grasp the stalk (7). The robotic arm (5) remains stationary.

Figure 4B shows the closed jaws (3) of the gripping unit withdrawn towards the harvesting unit (10), while the clamping jaws (2) and the cutting elements (1) remain open. The stalk (7) is positioned between the jaws (3). The closing of the gripping jaws signals the next working step, in which the robot arm (5) is moved together with the harvesting head (10) downwards and backwards (away from the fruit) to generate a reaction on the arms of the gripping jaws (3). The pressure of the upper surface of the cup sepal (9) (Figure 4C) of the strawberry fruit (8) on the gripping jaws (3) bends them, causing the sensor contacts (4) to short-circuit. This generates a control signal that stops the arm movement and causes the jaws (2) to clamp on the stalk (7) to immobilise the fruit. Closing the clamping jaws triggers the cutting unit (1). When the cutting unit closes, the arm (5) moves over the container.

The gripping, clamping, and cutting mechanism has an elongated design to facilitate reaching the fruit even inside the strawberry plant. In addition, the extendable stalk gripping unit increases its working range, making it possible to reach the fruit inside the plant. The use of a sensor (4) makes it possible to determine a fixed length of the stalk and to obtain a signal for the automatic control of the robotic arm and other components of the harvesting unit, which contributes to reducing the operation time.

**Figure 5:**
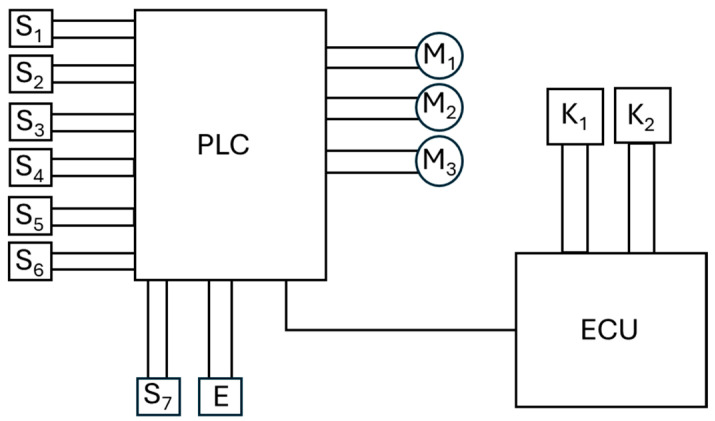
Block diagram of the gripper control system: S_1_, S_2_—contacts for opening the gripping element; S_3_, S_4_—contacts for opening the clamping element; S_5_, S_6_—contacts for opening the cutting element; S_7_—contact for determining the appropriate length of the stalk (deflection of the arms of the gripping element); E—electromagnet pulling in the gripping element; M_1_, M_2_, M_3_—motors; K_1_, K_2_—cameras determining X, Y, Z coordinates; ECU—Electronic Control Unit; PLC—Programable Logic Controller.

Figure 5 shows a block diagram of the connection of microswitches, which act as limit switches S_1_–S_6_, and motors M_1_–M_3_, which control the screw mechanisms of the individual jaws of the harvesting head, to the programmable logic controller (PLC). The gripping unit is equipped with microswitches S_1_–S_2,_ the clamping unit with microswitches S_3_–S_4_, and the fruit cutter with microswitches S_5_–S_6_. Microswitch S_7_ (see Figure 3, item 4) serves as a position sensor for the clamping unit, determining the height of the fruit cut (the length of the stalk), and is additionally equipped with a pull-in electromagnet E for the reciprocating motion of the gripping unit. The microswitches in the limit switch system precisely protect the mechanics of the harvesting assembly from exceeding the permissible opening, or closing angle of the jaws. The PLC is connected to the main control computer ECU (Electronic Control Unit) via a data bus, to which the vision system of cameras K_1_ and K_2_ is also connected. The vision system is responsible for scanning and determining the position (X, Y, Z) of the fruit to be harvested. In addition, the ECU manages the operation of the mobile robotic arm on which the strawberry harvesting head is mounted.

**Figure 6:**
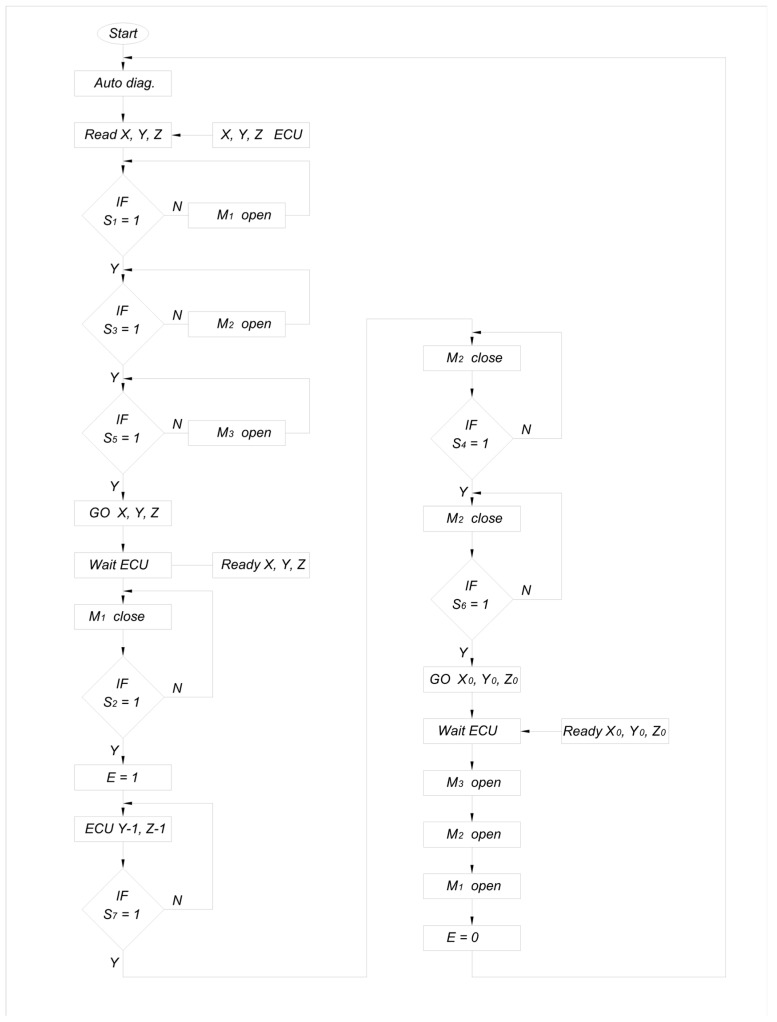
Control algorithm for the gripper head.

The phases of the strawberry fruit harvesting cycle are shown by the algorithm presented in Figure 6. 

The gripper performs the following phases of work:

- First Phase of Harvesting: This phase consists of checking the correctness of the diagnostic parameters, including signals from microcontacts S_1_–S_6_ and the resistance of motors M_1_–M_3_ and electromagnet E (self-diagnostics).
- Second Phase: This phase involves reading the X, Y, Z coordinates calculated from the image and, in several steps, checking the opening of the jaws of the working elements. A signal appears on the contacts of the gripping jaws (S_1_), clamping jaws (S_3_), and cut-off element (S_5_). If opening is necessary, the appropriate motors (M_1_–M_3_) are started. After opening, the gripping jaw will move forward (with electromagnet E = 0, the spring moves the jaws forward) so that they protrude beyond the clamping jaws and the cut-off element.
- Third Phase: This phase involves closing the jaws of the gripping unit, indicated by a signal from sensor S_2_ obtained through the activation of motor M_1_. The fruit stalk (7) will be positioned between the closed jaws of the gripping unit, while the gripping jaws and cutting elements remain open (S_3_ and S_5_). The head is then ready to retract the gripping jaws and move the entire gripping assembly in the Y-1, Z-1 planes until it receives a signal (S_7_) from the sensor indicating that the set stem length has been achieved.
- Fourth Phase: This phase is the retraction of the gripping jaws (activation of the electromagnet E = 1) to align with the clamping jaws. The next step is to move the arm, on which the head is mounted, in the Y-1, Z-1 planes. The flower (9), resting on the lower part of the gripping jaw arms, causes the gripping arms to rotate on the swing joint (11) until the S_7_ sensor contacts close (signal). This signal is used to stop the movement of the robot arm and proceed to the next phase.
- Fifth Phase: In this phase, the jaws of the pressing unit close, indicated by signal S_4_, resulting in gripping of the stem. The cutting element then closes, triggered by the signal from contact S_6_, cutting the strawberry at the designated place on the stem.
- Sixth Phase: This phase involves the closed gripping jaws and closed cutting elements. The control system commands the head to displace to the coordinates X_0_, Y_0_, Z_0_, where a collection container for the fruit is located.
- Seventh Phase: After reaching the position above the collection container, the jaws of the clamping unit open, and the M_2_ engine runs until the S_3_ signal is obtained, allowing the fruit to be placed in the container. The cutting element (S_1_ = 1) and the gripping element (S_3_ = 1) are then opened. Finally, the arm moves the harvesting unit to the next position, where a strawberry was found to be picked, at a new X, Y, Z position.

The M_1_ motor activates the following contacts: S_1_ = 1 and S_2_ = 0 for gripping jaws open, S_1_ = 0 and S_2_ = 1 for gripping jaws closed. The M_2_ motor activates the following contacts: S_3_ = 1 and S_4_ = 0 for crimping jaws open, and S_3_ = 0 and S_4_ = 1 for crimping jaws closed. The M_3_ motor activates the following contacts: S_5_ = 1 and S_6_ = 0 for cutting scissors open, S_5_ = 0 and S_6_ = 1 for scissors closed. The electromagnet works in mode 0 or 1. E = 1 means that the electromagnet is activated and that the gripping jaws are retracted, while E = 0 indicates that the power is turned off, which causes the gripping jaws to extend.

The gripping, clamping, and cutting-off mechanisms described briefly have design proportions that give them an elongated shape, facilitating easier access to the fruit even inside the strawberry bush. In addition, the extendable stalk gripping unit makes the design of the picking unit slimmer in the early stages of harvesting, while the gripping and cutting units do not interfere with bush penetration. The use of a fruit displacement sensor provides an automatic signal to control the robotic arm and other components of the harvesting assembly. As a result, the stalk can be captured over a wide range of distances from the fruit without adversely affecting the point at which the fruit is cut from the stalk. The sensor’s operation will contribute to a significant repeatability in the length of the stalk cut off with the harvested fruit, maintaining a predetermined value. This will also improve the quality of harvested strawberry fruit. Furthermore, the use of the fruit displacement sensor can contribute to reducing the operation time, as it makes it easier to position the harvester in relation to the fruit to achieve the correct length of the cut stalk.

## 4. Structural Form

**Figure 7:**
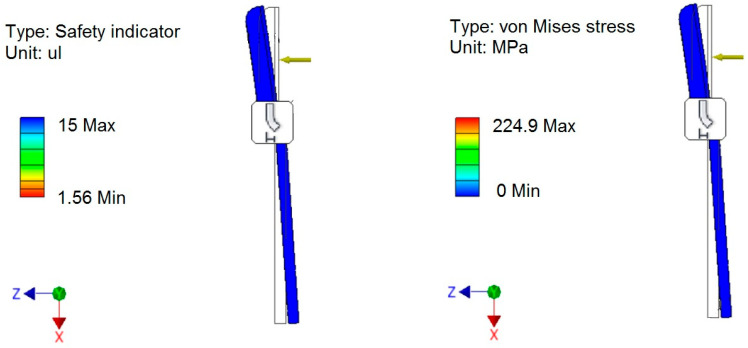
Safety factors and stress in the cutting elements.

**Figure 8:**
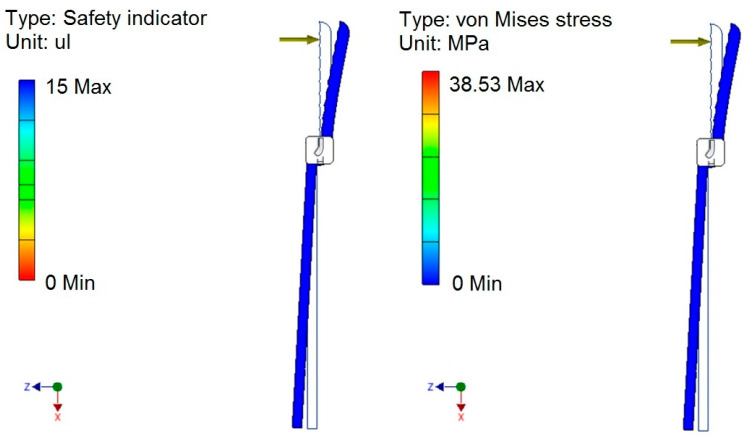
Safety factors and stress in the clasping jaws.

To reduce product development time and limit the number of prototypes, we utilized the Finite Element Method in Inventor software to conduct strength calculations. This approach enabled us to determine the stress values and safety factors for the most heavily loaded structural elements. The results of the stress simulations caused by cutting strawberry peduncle are presented in Figure 6, Figure 7 and Figure 8. The cutting force (F_2_) shown in Figure 7 was empirically measured at approximately 7 N, while a value of 8.5 N was used for the simulation, representing a 20% safety margin for the structure. The stresses resulting from cutting remained within acceptable lower ranges. Furthermore, the safety factors for the cutting mechanism were at the maximum levels, demonstrating the high strength of the cutting elements made from carbon steel.

The values of the safety factors and stresses simulated on the clasping jaws are presented in Figure 8. These values have been determined with aluminium as the construction material for the clasping mechanism. The simulation was conducted based on an adopted clasping force of 5 N, applied at the midpoint of the clasping jaw length. The calculated stresses occurring in the clasping jaws fell within the lower range of stress values, while the safety factor was in the high range.

**Figure 9:**
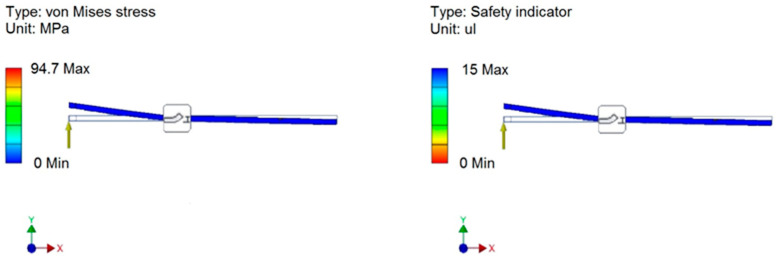
Factors of safety and stress in the clasping element.

Aluminium was chosen as the construction material for the clasping jaws, which are designed to grasp the fruit and extract it from the plant. Next, they send a request to the system to determine the required length of the peduncle. Consequently, the clasping forces were not assessed for this element; instead, the forces acting during the determination of the correct length of the peduncle were analysed. The results of the simulation tests regarding stresses and the safety factor in the jaws clasping the peduncle are shown in Figure 9. The relationship between the sensor triggering force and the displacement from the zero position is illustrated in Figure 13a. A force of 1 N (F_3_) was adopted for the simulation, as this represents the maximum force value determined experimentally, which is 0.6 N. Based on computer calculations, it can be concluded that these safety factors and stresses do not pose any threat to the structure.

The values obtained for stresses and safety factors indicate the potential for optimising (slimming down) the final structure of the fruit harvesting unit. In conclusion, we suggest that the cutting mechanism should be made of carbon steel, while the clasping jaws should be made of aluminium.

Physical verification of the model was conducted under laboratory conditions. For this purpose, strawberries grown in the laboratory on a coconut substrate were utilised, simulating the growing condition in NFT tunnels.

**Figure 10:**
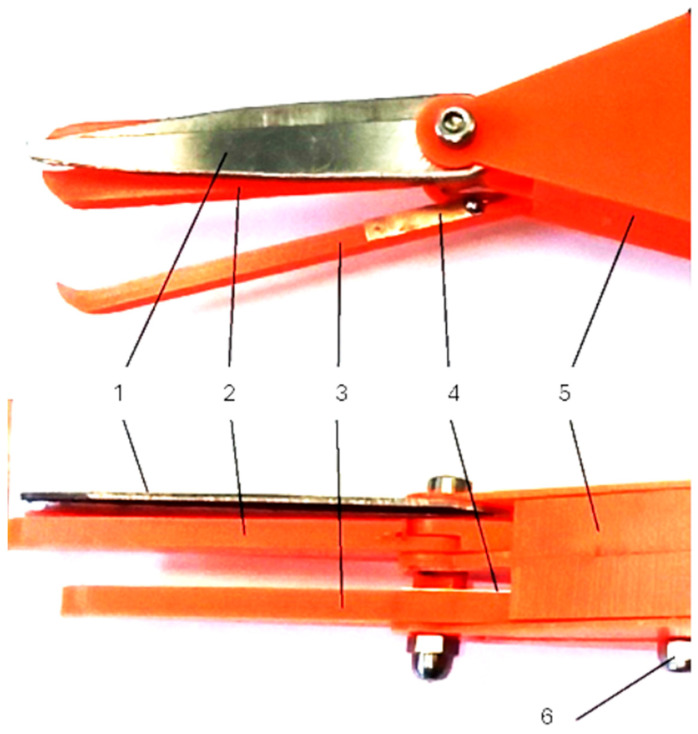
The prototype of the harvesting assembly under study: 1—cutting elements, 2—clamping jaws, 3—gripping jaws, 4—sensor determining the length of the stalk, 5—body of the collecting assembly, 6—swinging and sliding attachment of the gripping jaws.

The tested prototype of the harvesting assembly, designed for strawberry fruit harvesting robots, is illustrated in Figure 10.

**Figure 11:**
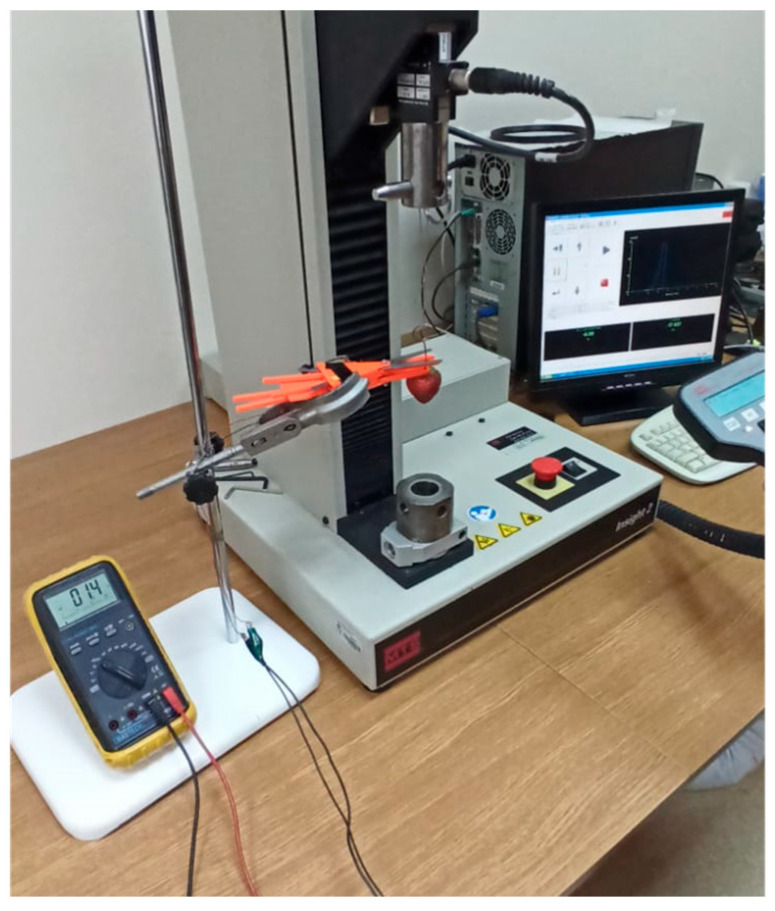
Test stand with the MTS testing machine.

The functional model of the harvesting unit (Figure 10) underwent laboratory tests aimed at determining the following parameters: the length of the peduncle that remains attached to the fruit after cutting, the triggering force required to activate the fruit movement sensor (indicated by the closure of contacts), and the cutting force necessary to sever the peduncle. The measurements were conducted using the MTS testing machine equipped with a strain gauge head, with a measuring range of up to 25 N (Figure 11). Additionally, the tests provided data on the weights and dimensions of the tested fruits (widths a and b, and height c).

**Figure 12:**
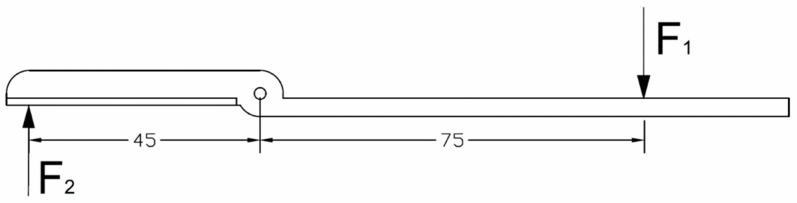
Distribution of forces on the cutting element.

The functional model of the harvesting unit was designed and manufactured based on the concept derived from the morphology table, with strength calculations performed using the finite element method. The prototype was developed as a fully functional model capable of harvesting fruit. The cutting elements were constructed from high-carbon steel (type D400) and subsequently tested to determine the cutting force required for effective stem cutting. The values of the cutting forces on the cutting element blade—measured at a distance of 45 mm from the axis of rotation—were established (Figure 12). The application of the cutting force at this position determines the maximum moment of resistance acting on the blade, which is influenced by the design and operation of the jaws gripping the peduncle. The indicated force F_1_ represents the measured pressure exerted by the cutting element, while force F_2_ corresponds to the peduncle cutting resistance. The lever mechanism depicted in Figure 12, with marked lengths of 75 mm and 45 mm, enhances the force F_2_ by approximately 40%.

**Figure 13:**
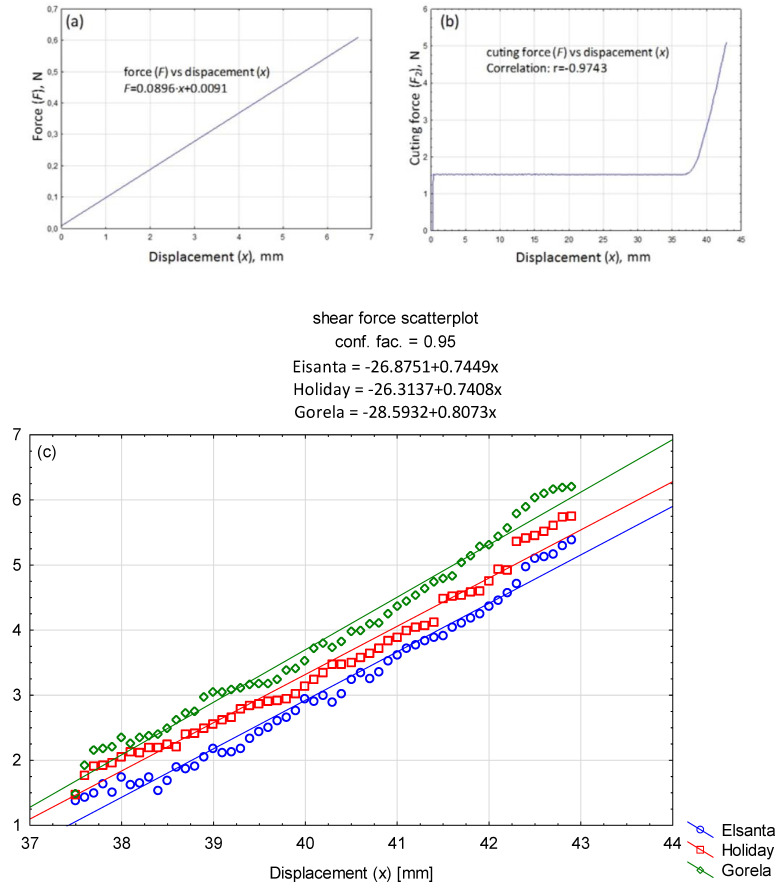
(**a–c**) The relationship between the triggering force of the strawberry fruit displacement sensor and the shear force as a function of the displacement of the jaws grasping the stalk.

The results obtained were statistically analysed using linear regression in the STATISTICA programme. A significance level of 95% for two degrees of freedom was assumed for the analysis. Regression analyses were carried out to identify substantively justified correlations between the studied quantities. Strawberries of three varieties—Elsanta, Holiday, and Gorella—were used in the study. The cutting force was measured using the MTS testing machine (Figure 11), which provided stable measuring conditions. The cutting speed of the specimen for all tests was set at 60 mm/min. A strain gauge sensor with a measuring range of 0–25 N, based on previously conducted tests, was utilized for the cutting force measurements. The cutting force was recorded continuously at 500 Hz throughout the entire cutting process of the specimen. For all measurements, the cutting diagram was rearranged, as shown in Figure 13, with the cutting phases clearly visible. The trigger force of the fruit displacement sensor (F) was also determined using the MTS device, with the same test settings applied as for the cutting force measurements.

The maximum triggering force of the fruit displacement sensor was found to be in the range of 0.45–0.60 N, as illustrated in Figure 13a. The average force that triggered the sensor in response to stress on the fruit peduncle, described as a function of the displacement (deviation) of the jaws gripping the peduncle, indicate that the average triggering force value was achieved after the end part of the gripping jaws had moved by 6–7 mm.

The fruit tissue was analysed for potential necroses caused by the excess breaking stresses resulting from the pressure exerted by the gripping jaws on the fruit. It was found that none of the tested fruits exhibited mechanical damage due to the force that triggered the sensor. No wet or dry bruises were identified after 24 and 72 h after the test.

The empirically determined value of the cutting force was approximately 7 N. The observed changes in the mean value of the forces acting on the peduncle as a function of displacement is characteristic for anisotropic materials. Figure 13c shows an enlarged section of the cut proper (Z) from Figure 13b.

## 5. The Length of the Peduncle after Cutting the Fruit

**Figure 14:**
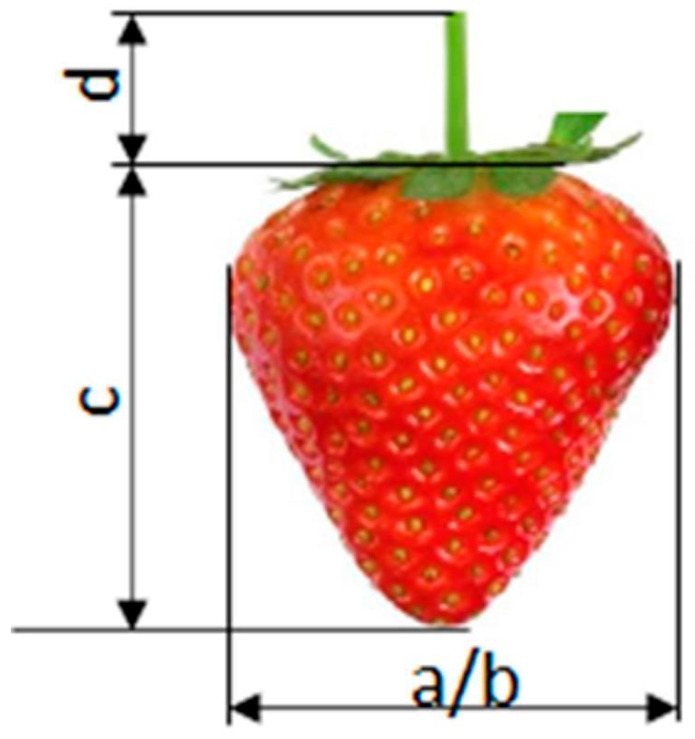
Fruit dimensions with determined peduncle length; a, b—diameter of the fruit, c—height of the fruit, d—length of the stalk.

**Figure 15:**
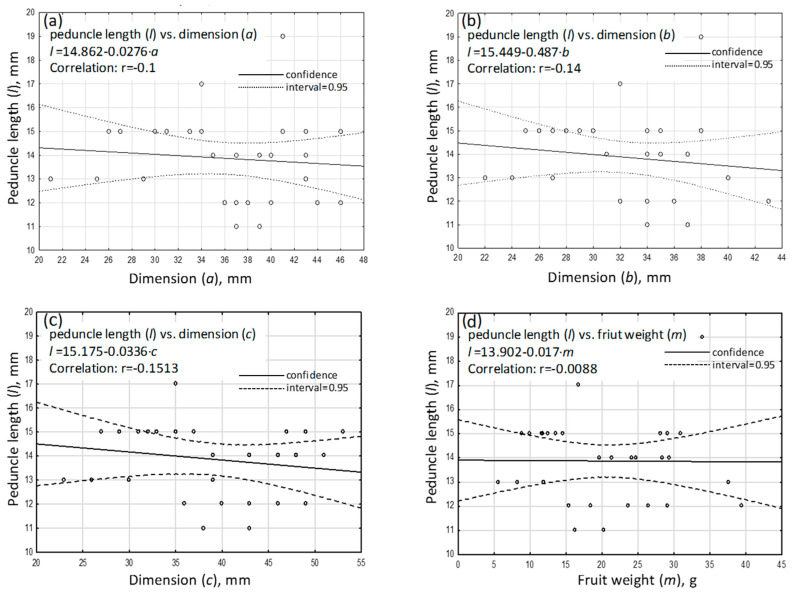
The relationships between various features and the length of the peduncle remaining on the fruit after harvesting: (**a**)—dimension a, (**b**)—dimension b, (**c**)—dimension c, (**d**)—weight m.

Figure 14 shows the taken-for-granted dimensions of the fruit including the peduncle. The results, shown in Figure 15a–d, indicate a lack of correlation between the fruit weight and dimensions a, b, and c and the length of the peduncle left on the fruit d. The obtained correlation coefficients between the peduncle length and the studied morphological parameters of the fruit proved to be statistically insignificant.

From the perspective of the proper operation of the harvesting head, the relationship observed between the length of the cut-off peduncle and the tested morphological parameters of the fruit is a positive indication. This is because all fruits will have a certain length of peduncle remaining after harvesting, with statistically comparable values that do not differ significantly, regardless of their weight and dimensions a, b, and c. The average length of the cut peduncle was 14 mm. The designated length of the remaining peduncle is determined by the design specifications of the head and falls within the recommended range of 5–20 mm. The results obtained from the tests confirm the repeatability of the operation of the harvesting unit.

## 6. Performance Analysis of the Robot Vision System for Strawberry Harvesting

**Figure 16:**
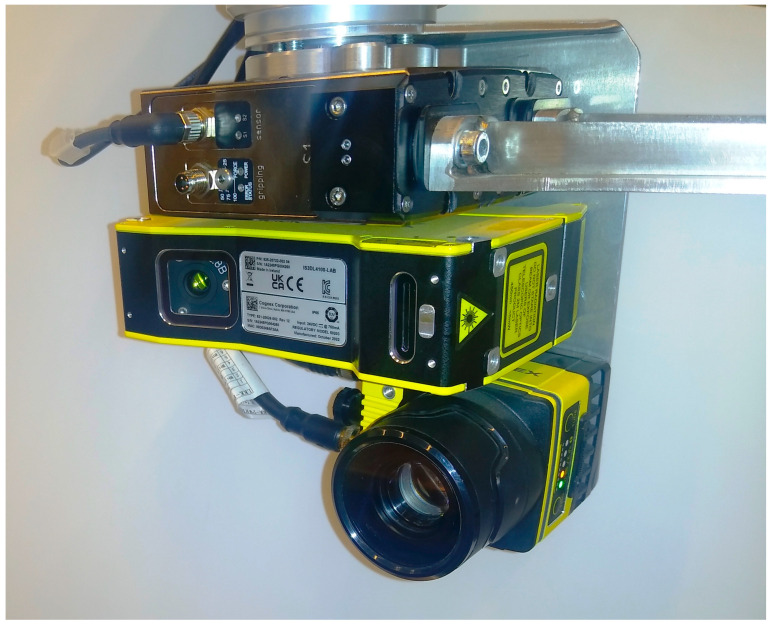
Robot end effector with visible vision system.

The objective of the research in this area was to configure a vision system for the robotic harvesting of strawberries in gutter cultivation. The vision system is responsible for identifying the fruit to be harvested and calculating and sending coordinates to the robotic manipulator, which positions the harvesting unit accordingly. Two x- and y-coordinates are determined from the 2D camera image, while a third z-coordinate is established using the camera as a 3D scanner. This coordinate reflects the distance of the strawberry from the axis that determines the direction of travel of the harvesting robot. Figure 16 shows a photograph of the robot’s complete effector, which includes the cameras of the vision system and the gripper.

The task set for the system was that the harvesting time for a single strawberry fruit should be less than 4 s; hence, the prototype uses cameras equipped with very fast image sensors. Since the vision system, robotic arm, gripper, and driving of the platform are controlled overhead by a PLC, efficient operation of the vision system is required to provide the most crucial data—specifically, where the strawberries ready for harvesting are located.

**Figure 17:**
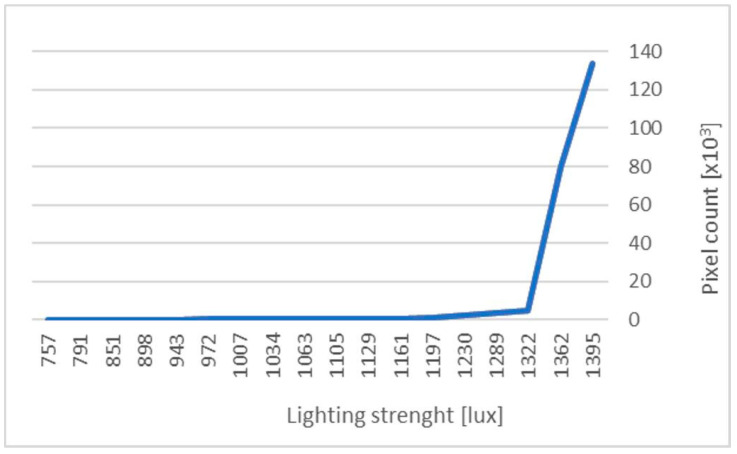
Relationship between light intensity and the number of identified red coloured pixels.

In the prototype under study, the robot’s vision system works in conjunction with an LED lamp, which serves to illuminate the area to be harvested in a single cycle. The working area of the illuminator is 0.096 m^2^, while the luminous flux value is 5700 lm. As part of the experimental study, measurements were carried out to determine the effectiveness of the vision system at different light intensity levels. The results, illustrated in a graph (Figure 17), show the relationship between the light intensity, expressed in lux, and the number of detected pixels corresponding to the red colour of a strawberry. The measurements were taken from a single shot covering the view of two fruits, maintaining a constant distance between the fruits and the camera, as well as consistent camera settings. The camera configuration was established based on prior trials and observations of the system’s performance.

**Figure 18:**
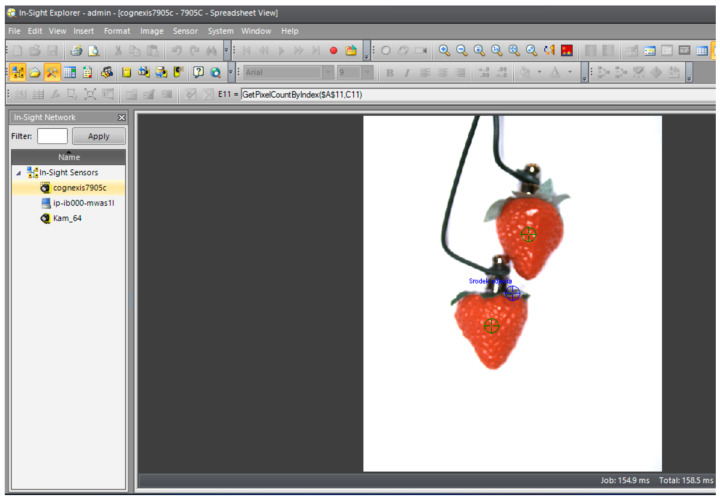
Recorded image of two strawberries in the tool software.

A photo of the camera image captured on the computer is presented in Figure 18. It shows two strawberry fruits supported by a rigid connection.

**Figure 19:**
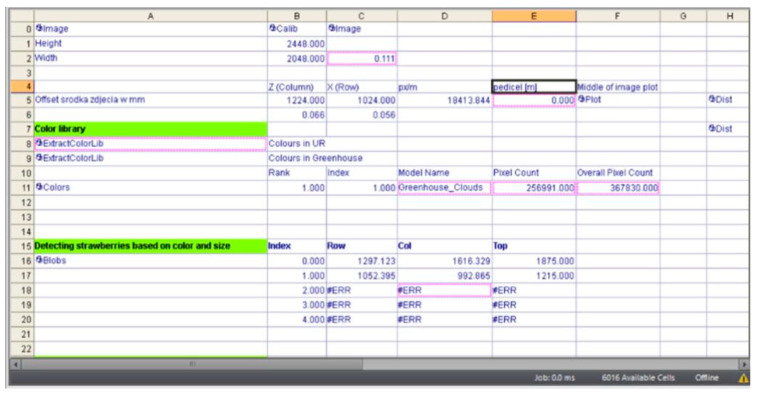
Summary data for the recorded image of strawberries—screenshot.

The data read by the software regarding the object detected by the camera are presented in the form of a table (Figure 19).

**Figure 20:**
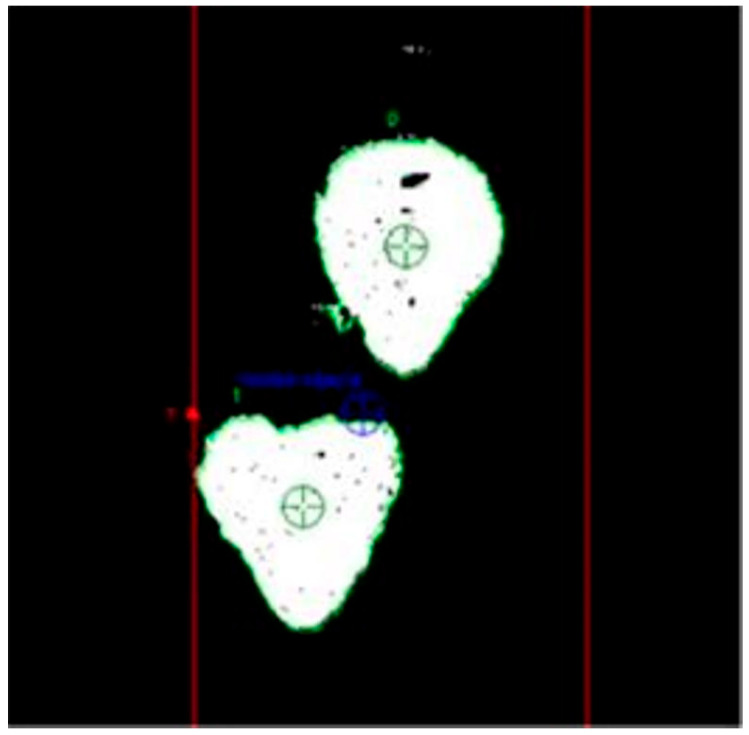
View of strawberry fruit with determined apparent centre of symmetry.

Row 11, titled ‘Colors’, contains information about the number of red pixels, while rows 16 and 17 provide coordinate values for capturing individual fruits by their stems. These coordinates are calculated using a programmed algorithm that takes into account the apparent centre of symmetry of the fruit, visible as a 2D planar object (Figure 20). The points of symmetry are indicated by green targets. The design of the gripper incorporates some compensation for errors in determining this point, which can affect the alignment of the petiole relative to the fruit.

**Figure 21:**
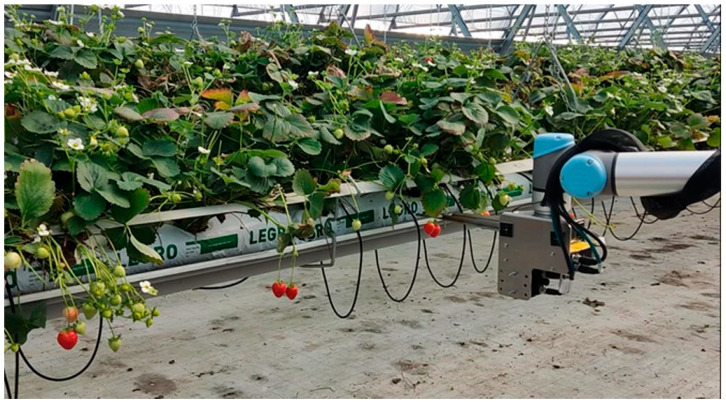
Harvesting strawberries with the robot.

Experimental harvesting tests conducted under real conditions (Figure 21) demonstrated a high accuracy of 95% in identifying ripe fruit, with the harvesting efficiency of the robotic arm reaching 90%. This indicates that only one fruit out of ten was not harvested, potentially due to the movement of previously identified fruits with fixed position positions (such cases were observed). 

## 7. Discussion

In their review, Vrochidou et al. [29] presented modern designs of grippers dedicated to the harvesting of various fruits and vegetables, concluding that grippers operating on the principle of grasping the product are most often used in robotic harvesting. For example, Roshanianfard and Noguchi [30] proposed a manually operated five-finger picker for pumpkin harvesting, while Taqi et al. [31] introduced the design of a two-finger gripper specifically for harvesting cherry tomatoes. They measured the force required to cut off the fruit and found, through experiments, that issues arose with securing the tomato stem in the jaws and extracting the harvested fruit. 

In terms of grapple design, Bac et al. [32] delineated the Fin Ray and Lip-type grapples for harvesting peppers. They determined the percentage of correctly cut fruit, depending on the density of the plant leaves, and discussed both the advantages and disadvantages of the robotic harvesting of peppers. Conversely, Zhou et al. [33] reported the results of a study on harvesting tomatoes using a flexible four-chamber finger picker. The authors calculated the pressure within the fingers based on the weight of the harvested fruit, ensuring it remained undamaged. 

In another study, Longtao et al. [34] employed a finger gripper fitted with sensors to optimise the gripper’s positioning for kiwifruit harvesting. The gripper’s fingers were equipped with pressure sensors, and the study demonstrated that this solution enabled non-destructive fruit harvesting. Li et al. [35] stated that future gripper innovations will include controlled rigid or flexible bionic manipulators, offering high efficiency, low damage to plants, and improved accuracy in identifying harvested fruits and vegetables.

Designs for crop harvesting grapple systems are not exclusively limited to those that grasp fruits or vegetables. For instance, Yeshmukhametov et al. [36] developed a grapple consisting of two semi-circular bowls with cutting knives installed on the bowl edges. The authors noted as a benefit of this design that leaving part of the stem on the harvested fruit prolongs the storage time. Zhao et al. [37] conducted a study utilising a robotic system for cherry tomato harvesting that employed a vacuum gripper housed in a cylindrical cup, along with a rotating cutting unit as the operational element. The authors concluded that this approach facilitated proper harvesting. A similar solution was explored by Jun et al. [10], who developed a tomato harvesting robot that combined a vacuum gripper with a scissor cutting unit. The authors noted, based on their experiments, that the developed design required modification due to difficulties in holding heavier tomatoes and the damage caused by the cutting unit.

Researchers have also experimented with various types of grippers for strawberry harvesting. For example, Xiong et al. [38] presented a finger picker featuring a cutting unit at the upper part of the jaws to detach the fruit from the stalk, with a container positioned at the lower part of the gripper to collect the fruit. The authors found that by handling the delicate strawberries properly, the designed structure ensured a stable harvesting process. In another study, Xiong et al. [14] improved the gripper by installing a cable drive to control the position of the fingers and adding a hopper positioning sensor. Field tests yielded satisfactory cycle times, yet harvesting inadequacies were attributed to the limitations of the vision system and insufficient dexterity of the gripper. 

De Preter et al. [39] presented the design of a rotary finger gripper attached to a self-propelled robotic arm. In their field tests, they achieved promising fruit harvesting times, but identified operational issues. The authors noted that the primary challenge was the gripper’s tendency to open due to obstacles such as leaves, twigs, and strawberry bunches, concluding that this issue could be resolved by installing an additional manipulator to separate them. Scissor end effectors are commonly used solely for fruit separation [14,40,41] or are equipped with additional vacuum devices to hold the fruit [42,43]. Contact grippers, often in the form of three-finger clamps with a force-limiting function, are also prevalent [44], as are two-finger or multi-finger heads that perform rotational movements to twist the stalk [45]. 

Kurpaska et al. [45] reported on the use of a vacuum gripper for strawberry harvesting, analysing three types of suction cups to ascertain the required suction force for effective grasping. They assessed the stress imparted on the fruit and the resulting area of deformation, observing the formation of haematomas on strawberry fruits caused by excessively high vacuum levels in the suction cups.

Online video resources display the operation of various grippers. Designers have proposed a jaw-type picker whereby the fruit is detached from the stalk by means of a belt [46] or through the rotation of the picker arms [44,45,46]. Another design [22,47] includes an integrated, rotating six-armed jaw system, with each arm equipped with two jaws serving as a single fruit picker. Notably, this system lacks a mechanism for cutting the fruit from the stalk; instead, harvesting involves clamping and constricting the upper part of the picking unit, ultimately detaching the strawberry from the stalk. 

Conversely, another design solution [23,24] proposes a pneumatic–mechanical system wherein an extendable suction cup grasps the fruit, which is then manoeuvred alongside the head with jaws move to secure the fruit. Once immobilised, the fruit is detached from the stalk by the rotational movement of the gripper about its axis. A design presented in additional studies [25,26,27,28] is grounded in steel scissor jaws that grip the peduncle before cutting off the fruit.

Contact grippers often risk crushing delicate strawberries [43] or causing haematomas on the fruit surface due to excessive contact force or a vacuum. An essential aspect leading to surface damage is the duration of the exerted force.

The design solution proposed by the authors of this article for the strawberry fruit-gripping head effectively mitigates haematoma formation, as the head does not make direct contact with the fruit during harvesting and allows for a predetermined length of the stalk. This stalk length can be tailored to align with consumer preferences or bulk purchasing requirements. The aforementioned harvesting guidelines cannot be met by current strawberry pickers or by most existing designs of gripper heads.

## 8. Conclusions

The analysis of strawberry head grippers presented in this article demonstrates a variety of designs that facilitate different harvesting approaches, primarily by gripping the fruit with the working elements. However, this method can lead to the occurrence of bruising on the fruit. The innovative solution introduced in this article features a novel head specifically designed for strawberry harvesting. Laboratory tests have shown that the new head does not cause any mechanical damage to the fruit, and the average length of the trimmed stalk is approximately 14 mm. This set stalk length is derived from the design parameters of the head and falls within the recommended range of 5–20 mm. The elongated shape of the grasping jaws, combined with the reciprocating motion of this component, enhances penetration into the strawberry bush and reduces the likelihood of fruit damage.

The results obtained confirm the reproducibility of the harvesting assembly. The analysis carried out suggest that further research should focus on material optimization and the development of a universal connection system compatible with various robotic arms and control systems. The incorporation of a microswitch system will facilitate process control during harvesting through an algorithm programmed in the PLC, ultimately leading to a fully automated gripper head. According to the authors, the implementation of a seven-stage harvesting process will enhance both the repeatability and precision of harvesting compared to existing solutions. 

The test results clearly indicate the potential for further development of the newly designed head and its control algorithm. The next phase of research should involve field tests to assess both the number of fruits harvested and the extent of any fruit damage incurred. It is noteworthy that this publication serves as an important source of information in this context.

## Data Availability

Data are contained within the article.
